# Short-term neonatal and long-term neurodevelopmental outcome of children born term low birth weight

**DOI:** 10.1038/s41598-024-52154-9

**Published:** 2024-01-27

**Authors:** Ho Yeon Kim, Geum Joon Cho, Ki Hoon Ahn, Soon-Cheol Hong, Min-Jeong Oh, Hai-Joong Kim

**Affiliations:** Department of Obstetrics and Gynecology, Korea University College of Medicine, 148 Gurodong-Ro, Guro-Gu, Seoul, 08308 Republic of Korea

**Keywords:** Health care, Medical research, Neurology, Risk factors

## Abstract

This study aimed to examine the impact of term LBW on short-term neonatal and long-term neurodevelopmental outcomes in children 5–7 years of age. This is a population-based cohort study that merged national data from the Korea National Health Insurance claims and National Health Screening Program for Infants and Children. The participants were women who gave birth at a gestational age of ≥ 37 weeks between 2013 and 2015 in the Republic of Korea, and were tracked during 2020 for the neurodevelopmental surveillance of their children. Among 830,806 women who gave birth during the study period, 31,700 (3.8%) of their babies weighed less than 2500 g. By Cox proportional hazard analysis, children aged 5–7 years who had LBW were associated with any developmental, motor developmental delay, cognitive developmental delay, autism spectrum, attention deficit hyperactivity disorders, and epileptic and febrile seizures.Children born with term LBW were more vulnerable to neurodevelopmental disorders at 5–7 years of age than those with normal and large birth weights. This study further substantiates counseling parents regarding the long-term outcomes of children being born underweight.

## Introduction

Birth weight indicates infant well-being and is a key factor in infant health policy. According to the World Health Organization, low birth weight (LBW) is defined as a birth weight of less than 2500 g irrespective of gestational age while fetal growth restriction(FGR) or small for gestational age (SGA) refers to estimated fetal weight or birthweight below the 10th percentile for gestational age^1^. The estimated incidence of LBW is more than 20 million infants worldwide^2,3^. The prevalence of LBW varies in low- and middle-income countries and could be as low as 2–3% or as high as 30%^2,4^. We recently conducted a study in collaboration with over 20 countries, examined the global pattern of LBW in an exclusive manner and found a varied distribution, with the highest incidence rate observed in Southwest Asia^5^. The main causes of LBW are premature birth and poor fetal growth. Yet mostly unidentified, chromosome abnormality, infection and placental dysfunction are major causes of poor fetal growth. Globally, infants born with LBW are susceptible to short- and long-term adverse health outcomes including neurodevelopmental disorders^6,7^. Studies have been inconsistent and inconclusive, but they emphasized that LBW children have a higher risk of developmental delay, lower cognitive and motor function, and more behavioral problems than normal birth weight children^8–12^. LBW adds to the public health burden^13^.

Understanding neurodevelopment in LBW children enables early and timely intervention for developmental delay, which could accelerate and improve health outcomes. Responsive stimulation during early life is crucial for later cognitive development in children^14^. Discussions on prematurity and LBW have been widely conducted, and while not fully established, causal relationships have been acknowledged. However, there is limited research on the association between the LBW of term-born infants and developmental outcomes. This population cohort study aimed to determine developmental outcomes including developmental delay, behavioral problems, and cognitive and motor performance in LBW infants compared to normal and large birth weight infants up to 7 years of age in South Korea.

## Materials and methods

### Data characteristics

This study was conducted by merging national data from the Korea National Health Insurance (KNHI) claims, National Health Screening Examination (NHSE), and National Health Screening Program for Infants and Children (NHSP-IC). In Korea, 97% of the population is enrolled in the KNHI program, and its claims database contains all their claims information. Therefore, this centralized database contains comprehensive information on diseases and their treatments except for non-insurance procedures. Using this database, we identified all pregnant women who delivered between January 1, 2013, and December 31, 2015. Subsequent developmental delay, motor developmental delay, cognitive developmental delay, autism spectrum disorder (ASD), attention deficit hyperactivity disorder (ADHD), and epileptic and febrile seizures in their children up to 5–7 years were tracked until December 31, 2020. The KNHI system provides an NHSP-IC linked to maternal data for all neonates. Key components of the NHSP-IC include a health examination of the children, assessment of their gestational age at delivery, and measurement of birth weight. This study was approved by the Institutional Review Board of the Korea University Medical Center (No. 2023GR0196) which waved the requirement for informed consent for the following reason. All information was provided for the study after it had been anonymized; therefore, informed consent was not obtained from the participants. All methods were performed in accordance with the declaration of Helsinki.

### Study population

**Figure 1:**
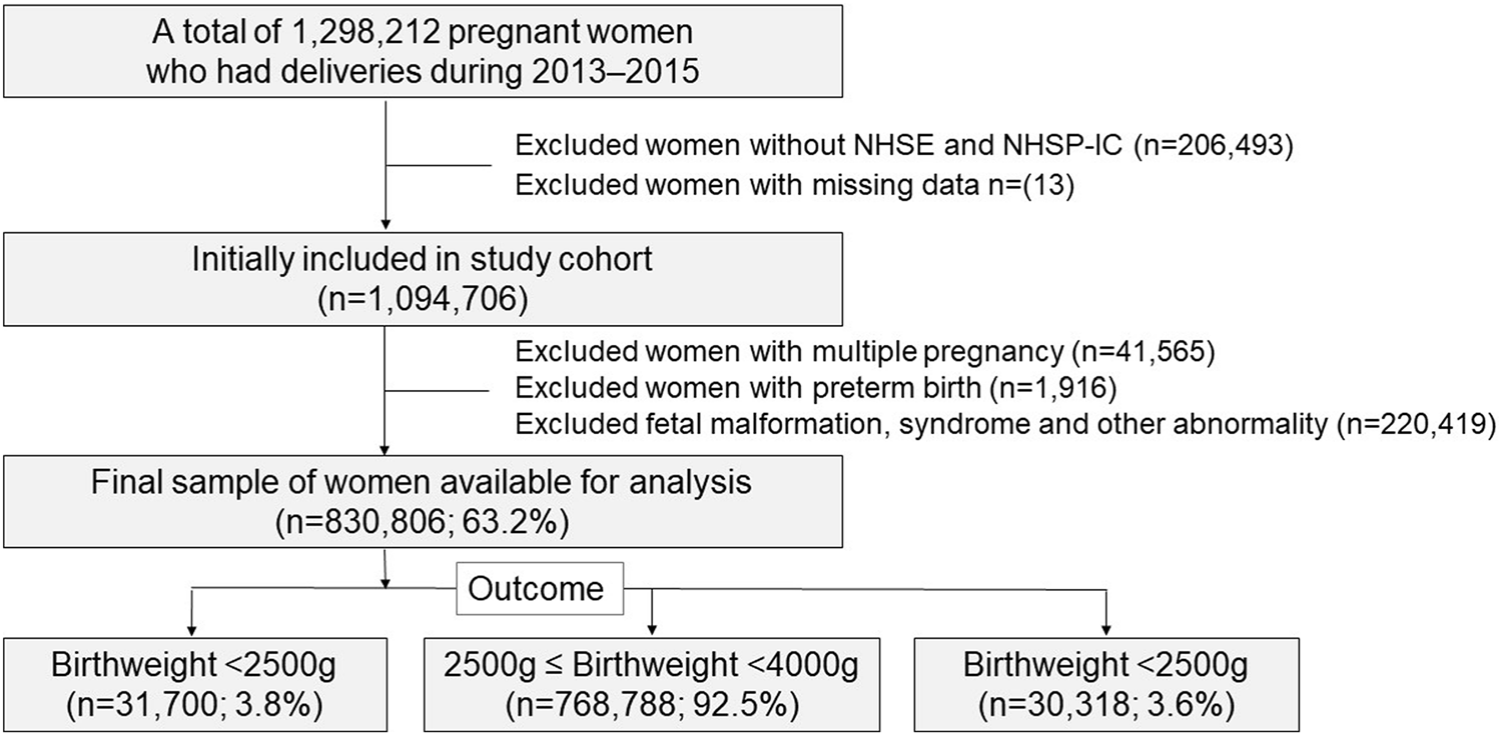
Flowchart of participant enrollment.

A flowchart of patient enrollment is shown in Fig. 1. Birth weight was categorized as LBW < 2500 g, normal birth weight 2500–3999 g, and large birth weight ≥ 4000 g. Women who had a gestational age of ≥ 37 weeks were included. Among these, women who had multiple pregnancies, preterm births, fetal malformations, syndromes and other abnormalities allocated Q code in the International Classification of Disease-10th Revision (ICD-10), women whose children did not undergo the NHSP-IC, and missing values were excluded. Perinatal factors were obtained on maternal characteristics (age, type of delivery, pregnancy-induced hypertension, gestational diabetes (GDM), hypertension and diabetes mellitus (DM) diagnosed before pregnancy) and infant characteristics (sex and birthweight) using the KNHI claims dataset. In addition, the medical issues of the infants including transient tachypnea, respiratory distress syndrome (RDS), necrotizing enterocolitis (NEC), intraventricular hemorrhage (IVH), bronchopulmonary dysplasia (BPD) and birth asphyxia were identified. Underlying maternal and neonatal diseases were identified according to the ICD-10 codes.

### Short-term outcomes of infants

Using the KNHI claims dataset, transient tachypnea, RDS, NEC, IVH, BPD and birth asphyxia were identified by principal or secondary diagnosis based on the codes of the ICD-10.

### Follow-up neurodevelopmental outcomes up to 5–7 years

Any developmental delay, motor developmental delay, cognitive developmental delay, ASD, ADHD, tics and stereotypic behavior, and epileptic and febrile seizures were identified by principal or secondary diagnosis based on the ICD-10 codes (Table 1).

**Table 1:** Diagnosis based on International Classification of Disease-10 codes.

Diagnosis	ICD-10
Transient tachypnea	P22.1
Respiratory distress syndrome	P22.0
Necrotizing enterocolitis	P77
Intraventricular hemorrhage	P52.0, P52.1, P52.2
Bronchopulmonary dysplasia	P27.1
Birth asphyxia	P21.9, P21.0, P21.2
Any developmental delay	F70, F71, F72, F73, F78, F79, F80.0, F80.1, F80.2, F80.8, F80.9, F81.0, F81.1, F81.2, F81.3, F81.8, F81.9, F82.0, F83.0, R26.0, R27.0, R48.0, R48.8
Motor developmental delay	F82.0, R26.0, R27.0
Cognitive developmental delay	F70.0, F71.0, F72.0, F73.0, F78.0, F79.0, F80.0, F80.1, F80.2, F80.8, F80.9, F81.0, F81.1, F81.2, F81.3, F81.8, F81.9, F83.0, R48.0, R48.8
Autism spectrum disorder	F84.0, F84.1, F84.4, F84.8
Attention-deficit/hyperactivity disorder	F90.0, F90.1, F90.2, F90.8, F90.9
Tics and stereotypic behavior	F95.0, F95.1, F95.2, F95.8, F95.9, F98.4, F98.5, R25.0
Epileptic and febrile seizures	G25.3, G40.0, G41.0, R56.0

### Statistical analysis

Continuous and categorical variables were expressed as mean ± standard deviation (SD) and percentages. Clinical and biochemical characteristics were compared among the groups using the t-test or one-way analysis of variance (ANOVA) for continuous variables and the χ2 test for categorical variables. The cumulative incidence of developmental delay, ASD, ADHD, tics and stereotypic behavior, and seizure and epileptic disorder was estimated using the Kaplan–Meier method and compared using the log-rank test. Cox proportional hazards models were used to estimate the adjusted hazard ratios (HRs) and 95% confidence intervals (CIs) for the rates of transient tachypnea, RDS, NEC, IVH, and BPD and for the development of developmental delay, ASD, ADHD, tics and stereotypic behavior, and seizure and epileptic disorder. Confounding factors adjusted were maternal age, undergoing cesarean section, pregnancy-induced hypertension, gestational diabetes, overt DM, hypertension before pregnancy, and the sex of the baby. Neonatal short-term complications were adjusted for analysis. Based on univariate analysis, variables with *p*-values < 0.2 were included in the Cox proportional hazards models. All tests were two-sided values, and *p*-values < 0.05 were considered statistically significant. Statistical analyses were performed using SAS for Windows, version 9.4 (SAS Inc., Cary, NC, USA).

## Results

### Characteristics of participants

The obstetric characteristics of the participants are presented in Table 2. Women with LBW and large birth weight babies were older and had a higher prevalence of undergoing cesarean sections than those with normal birth weight babies. The rates of pregnancy-induced hypertension and hypertension before pregnancy were significantly higher in women with LBW babies than in those with large or normal birth weight babies. Women with large birth weight babies had significantly higher rates of gestational and overt diabetes than those with normal or LBW babies. Male sex was more prevalent in large birth weight babies than normal or LBW babies.

**Table 2:** Pre-pregnancy and pregnancy characteristics.

	< 2500 g (n = 31,700)	2500 ≤ < 4000 g (n = 768,788)	4000 g ≤ (n = 30,318)	*p*-value
Age (years)	32.2 ± 4.5	31.9 ± 4.2	32.4 ± 4.1	< 0.001
Pregestational diabetes (%)	466 (1.5)	8,034 (1.1)	687 (2.3)	< 0.001
Gestational diabetes (%)	2,380 (7.5)	49,631 (6.5)	3,241 (10.7)	< 0.001
Cesarean section (%)	14,707 (46.4)	285,334 (37.1)	15,150 (50.0)	< 0.001
HTN (%)*	495 (1.6)	4,431 (0.6)	249 (0.8)	< 0.001
Pregnancy-induced HTN (%)	1,643 (5.2)	7,652 (1.0)	370 (1.2)	< 0.001
Male gender of baby (%)	14,120 (44.5)	380,288 (49.5)	18,920 (62.4)	< 0.001
Birth weight (kg)	2.08 ± 0.41	3.21 ± 0.33	4.68 ± 2.59	< 0.001

DM, diabetes mellitus; HTN, hypertension.

*HTN before pregnancy.

### Short-term complications in neonates

Cox proportional hazards models were used to estimate the adjusted HRs (aHRs) and 95% CIs for the development of short-term neonatal outcomes related to birth weight after adjustment for confounding factors (Table 3). Neonates with LBW had significantly higher rates of transient tachypnea (aHR 2.34, 95% CI 2.19–2.49), RDS (aHR 8.81, 95% CI 8.37–9.27), NEC (aHR 10.61, 95% CI 7.69–14.6), IVH (aHR 20.99, 95% CI 17.58–25.1), BPD (aHR 115.2, 95% CI 84.1–157.9), and birth asphyxia (aHR 3.52, 95% CI 2.82–4.39) than those with normal birth weight. Neonates with large birthweight had more transient tachypnea (aHR 1.54, 95% CI 1.43–1.66), IVH (aHR 1.62, 95% CI 1.02–2.59), and BPD (aHR 3.79, 95% CI 1.79–8.03) than those with normal birth weight.

**Table 3:** Neonatal outcome of LBW, normal birth weight and large birth weight.

	< 2500 g	2500-3999 g	4000 g ≤	< 2500 g	2500-3999 g	4000 g ≤
	N (%)			Adjusted		
Transient tachypnea	1089 (3.44)	11,028 (1.43)	743 (2.45)	2.339 (2.194–2.494)	1	1.538 (1.426–1.659)
RDS	2219 (7)	6044 (0.79)	293 (0.97)	8.811 (8.371–9.274)	1	1.109 (0.986–1.248)
NEC	57 (0.18)	121 (0.02)	2 (0.01)	10.608 (7.689–14.635)	1	0.385 (0.095–1.559)
IVH	239 (0.75)	270 (0.04)	19 (0.06)	20.994 (17.581–25.069)	1	1.623 (1.017–2.588)
BPD	241 (0.76)	47 (0.01)	8 (0.03)	115.225 (84.058–157.947)	1	3.788 (1.787–8.03)
Birth asphyxia	95 (0.3)	589 (0.08)	31 (0.1)	3.517 (2.82–4.386)	1	1.114 (0.775–1.601)

*Adjusted for age, GDM, overt DM, HTN, pregnancy induced HTN, cesarean section, gender of baby.

RDS respiratory distress syndrome, NEC necrotizing enterocolitis, IVH intraventricular hemorrhage, BPD bronchopulmonary dysplasia.

### Neurodevelopmental outcome stratified by birth weight

**Figure 2:**
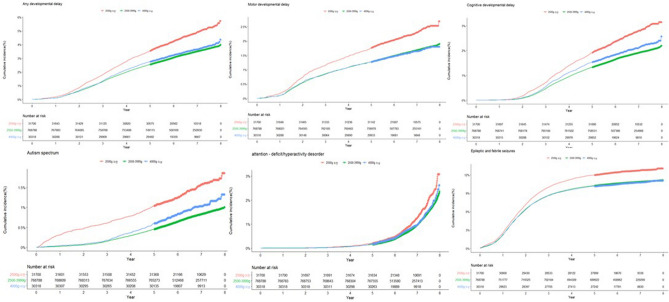
Kaplan–Meier curves for the cumulative incidence of cognitive, motor, and any developmental delays, ASD, ADHD, and epileptic and febrile seizures among the groups. ASD, autism spectrum disorder; ADHD, attention deficit hyperactivity disorder.

The neurodevelopmental outcomes related to the birth weight of the neonates were estimated using Cox proportional hazards models after adjustment for confounding factors (Table 4). LBW was associated with any developmental delay (aHR 1.36, 95% CI 1.29–1.44), motor developmental delay (aHR 1.31, 95% CI 1.22–1.42), cognitive developmental delay (aHR 1.43, 95% CI 1.33–1.54), ASD (aHR 1.76, 95% CI 1.59–1.95), ADHD (aHR 1.30, 95% CI 1.17–1.46), and epileptic and febrile seizures (aHR 1.14, 95% CI 1.10–1.18). There was no difference in neurodevelopmental outcomes between neonates with large and normal birth weights. Figure 2 shows Kaplan–Meier curves for the cumulative incidence of neurodevelopmental outcomes among the low, normal, and large birth weight groups. Up to 7 years of age, the cumulative incidences of motor, cognitive, and any developmental delays, in addition to ASD, ADHD, and epileptic and febrile seizures were significantly higher in the LBW group than in normal and large birth weight groups.

**Table 4:** Neurodevelopmental outcome of LBW, normal birth weight and large birth weight.

	< 2500 g	2500-3999 g	4000 g ≤	< 2500 g	2500-3999 g	4000 g ≤
	N (%)			Adjusted		
Any developmental delay	1,461 (4.6)	25,338 (3.3)	1055 (3.5)	1.366 (1.294–1.442)	1	0.97 (0.912–1.032)
Motor developmental delay	696 (2.2)	12,227 (1.6)	476 (1.6)	1.317 (1.218–1.425)	1	0.967 (0.882–1.06)
Cognitive developmental delay	821 (2.6)	13,703 (1.8)	605 (2)	1.439 (1.339–1.546)	1	0.971 (0.895–1.054)
Autism spectrum disorder	451 (1.42)	5728, (0.75)	287 (0.95)	1.758 (1.59–1.94)	1	0.982 (0.899–1.073)
ADHD	346 (1.09)	6476 (0.84)	275 (0.91)	1.307 (1.17–1.46		0.924 (0.819–1.043)
Tics and stereotypic behavior	322 (1.0)	7,189 (0.9)	288 (0.9)	1.101 (0.984–1.233)	1	0.937 (0.833–1.055)
Epileptic and febrile seizures	3,993 (12.6)	82,686 (10.8)	3,229 (10.7)	1.14 (1.104–1.178)	1	0.966 (0.933–1.001)

*Adjusted for age, GDM, overt DM, HTN, pregnancy induced HTN, cesarean section, gender of baby, transient tachypnea, RDS, NEC, IVH, BPD.

*ADHD attention deficit/hyperactive disorder.

## Discussion

This study examined the short- and long-term outcomes, including neurodevelopmental disorders, of babies with low, normal, and large birth weights using national population data. Children with LBW were not only susceptible to short-term complications but also suffered increasingly from motor, cognitive, and any developmental delays, ASD, ADHD, and epileptic and febrile seizures up to the age of 7.

LBW is a risk factor for neonatal morbidity and mortality, overall health, and developmental disorders^15^. However, previous studies have predominantly focused on the impact of LBW due to preterm birth, indicating that prematurity itself is a major cause of developmental disorders^16^, and research on LBW infants born at term has focused on IQ, learning, and behavior^17,18^. In addition, the definitions of small for gestational age (SGA) or fetal growth restriction, children age at assessment and outcome variables were varied with or without deficits in cognitive and learning abilities, and occurrence of attention problems^19^. Few studies have targeted the Asian population in this aspect.

Consistent with our findings, a Norwegian population study recently demonstrated a highly significant dose–response association between birthweight and cerebral palsy, vision/hearing disability, intellectual impairment, schizophrenia, epilepsy, ASD, and behavioral disorders such as ADHD^20^. Sacchi et al. performed a meta-analysis of 2230 children born at term who had intrauterine growth restriction or were SGA and demonstrated that they had lower cognitive scores than those who were appropriate for gestational age^15^. However, the other studies demonstrated no differences or mixed results in developmental outcomes, including school performance, attention problems, and psychological symptoms^21–23^. This could be attributed to methodological limitations due to multiple contributing factors, such as different ethnic backgrounds, social interactions, and economic aspects. Given the impact of ethnic differences on LBW infants, our study’s specific focus on the Asian population is of particular importance.

Poor growth, maternal malnutrition, poverty, stress, infections such as malaria and HIV, diarrhea, environmental toxins, psychosocial factors such as learning opportunities, caregiver interaction, violence, and maternal depression can contribute to the deterioration child brain functioning^24,25^. However, the exact physiological mechanisms behind LBW and its effects on development are not yet fully understood due to the complexity of the causal pathways involved. One possible theory is related to fetal programming. Abnormal fetal growth is a sign of substantial alterations in fetal programming. The fetus adapts and survives in the uterus by slowing its growth. Defective placentation is a phenomenon that results in poor fetal growth, and may cause hypoxemia, inflammation, undernutrition, and endocrine dysregulation, which could impair normal brain development^25,26^. It could also lead to chronic hypoxia, which causes poor growth and consequently cerebral injury, especially in the primary sensory and forebrain motor systems, resulting in cognitive, motor, and attentional deficits^27^. Barker's theory supports the concept of fetal programming, indicating that individuals born under conditions of inadequate nutrition during pregnancy face a higher risk of ischemic heart disease and mortality as adults^28^. This evidence has been further expanded in recent years, suggesting that children born in suboptimal conditions in the uterus may experience increased health issues.

Cerebral microstructural and metabolic changes were evident in fetal brain magnetic resonance imaging of LBW infants, and this might cause abnormal neurodevelopment^29^. Placental dysfunction, a situation which the fetus has a limited transport of nutrients and oxygen also explains the association between LBW and ASD. Consistent with our finding that the ASD rate was significantly increased in LBW infants, a meta-analysis including more than 8 million participants demonstrated a significant association between infants who were SGA and the risk of ASD^30^.

Morbidities associated with LBW such as RDS and BPD may cause poor neurodevelopment^31,32^. Hypoxia caused by short-term respiratory problems in the neonatal period can interfere with normal brain development. As demonstrated by our data, LBW infants are more likely to suffer from complications during the neonatal period owing to the suboptimal oxygen levels that can damage their rapidly growing neurons and prevent them from forming new connections. Despite our analysis adjusting for neonatal complications, there was still an increased rate of developmental delay in LBW infants.

There have been limited studies on the association between LBW and tics and stereotypic disorders, which are highly complex and multifactorial in etiology. A study on the Korean population reported associations between tics and perinatal factors and revealed no association with LBW^33^ which is consistent with our findings. However the age of onset of tics and stereotypic disorders is usually 4–6 years, and since our study included children aged 5–7 years, there are limitations in establishing an association between LBW and tics and stereotypic disorders.

Our study findings on the relationship between LBW infants born on term and developmental outcomes prompt the identification of risk factors and early intervention. Several modifiable factors in the early environment may have long-term effects on health and cognitive function. In a British study, the learning environment (parental reading and interest in education) influenced cognitive development independent of birth weight and social background^34^. A prospective study suggested that stimulation at home may improve neurodevelopment in LBW children, especially during the early period^35^. According to a study on Korean adopted children, developmental outcomes may differ within the first 2 years depending on environmental factors and nutritional supply alone^36^. This emphasizes the significance of the early identification of children who are at risk of cognitive and motor deficits, closely monitoring their development and intervening promptly and effectively to enhance their developmental abilities.

Parents typically do not consider their LBW infants born on term to be at a risk of developmental disadvantages, and their postnatal care is underestimated. Therefore, it is crucial for healthcare professionals to educate parents about the importance of early screening and evaluation of these infants to ensure their healthy development.

This study boasts several notable strengths, primarily deriving from a population-based cohort, which offers the advantage of comprehensive and extended follow-up. Furthermore, to mitigate potential sources of bias, the study meticulously identified and controlled for numerous underlying risk factors pertaining to perinatal characteristics. Nonetheless, it is imperative to approach our findings with a degree of caution, recognizing several limitations. First and foremost, the neurodevelopmental outcomes of the children under scrutiny in this research were determined based on ICD-10 codes extracted from insurance claims data. Consequently, questions regarding the reliability and accuracy of these diagnoses within this database may arise detailed diagnostic methods used for developmental delay have not been reviewed. However, a recent study showed a 78.1–88.7% sensitivity and 100% specificity when comparing the diagnoses obtained from insurance claims data with the verified diagnoses documented in patient medical records^37^. Furthermore we encountered limitations in accessing essential information regarding influential factors of growth and neurodevelopment, such as socioeconomic status, cognitive and motor stimulation during early life, parent BMI and education, social environment, and nutritional status after birth. Prospective studies considering these factors are needed to further investigate the association between birth weight and neurodevelopmental outcomes.

In summary, the findings of this study show that developmental delay, ASD, ADHD, and epileptic and febrile seizures were increased in LBW infants born at term. These findings support the notion that LBW infants born at term need timely evaluation of their neurodevelopment because early detection and intervention are key to improved quality of life. The findings of this study need to be confirmed in a prospective study. Furthermore, studies on the mechanisms involved in neurodevelopment associated with LBW should be conducted.

## Data Availability

The dataset generated during and analyzed during the current study are not publicly available due to dataset owned by government but are available from the corresponding author on reasonable request.

## Abbreviations

ADHD: Attention deficit hyperactivity disorder
ASD: Autism spectrum disorder
BPD: Bronchopulmonary dysplasia
CI: Confidence interval
HR: Hazard ratio
IVH: Intraventricular hemorrhage
NEC: Necrotizing enterocolitis
NHSP-IC: National Health Screening Program for Infants and Childre
RDS: Respiratory distress syndrome
GDM: Gestational diabetes
ICD-10: International Classification of Diseases-10th Revision
KNHI: Korea National Health Insurance
LBW: Low birth weight
NHSE: National Health Screening Examination
SGA: Small for gestational age
